# Mortality associated with third-generation cephalosporin resistance in Enterobacterales bloodstream infections at eight sub-Saharan African hospitals (MBIRA): a prospective cohort study

**DOI:** 10.1016/S1473-3099(23)00233-5

**Published:** 2023-07-13

**Authors:** Alexander M Aiken, Andrea M Rehman, Marlieke EA de Kraker, Lola Madrid, Meron Kebede, Appiah-Korang Labi, Noah Obeng-Nkrumah, Brian Nyamwaya, Eunice Kagucia, Derek Cocker, Kondwani Kawaza, Rebecca Lester, Kenneth C Iregbu, Nubwa Medugu, Philip I Nwajiobi-Princewill, Angela Dramowski, Tolbert Sonda, Asia Hemed, Sombo Fwoloshi, David Ojok, J Anthony G Scott, Andrew Whitelaw, for the MBIRA study collaborators, Jabir Aliye, Nega Assefa, Dumessa Edessa, Joseph Oundo, Mulu Berihun, Thomas Dankwah, Mary M Osei, Maud Fandoh, Margaret Gakpo, Caroline Mulunda, Benedict Mvera, Mabvuto Chimenya, Nicholas Feasey, Jane Mallewa, Khadija Abdulraheem, Tobechi A Akujobi, Chinelo H Okonkwo, Luzell Britz, André NH Bulabula, Aaqilah Fataar, Blandina T Mmbaga, Neema Ng’unda, Uchizi Chirwa, Nyambe Kakula, Charles Muntemba, Ruth Nakazawe

**Affiliations:** Infectious Disease Epidemiology Department, London School of Hygiene & Tropical Medicine, London, UK; Infection Control Program and WHO Collaborating Center on Patient Safety and Antimicrobial Resistance, University of Geneva Hospitals and Faculty of Medicine, Geneva, Switzerland; Infectious Disease Epidemiology Department, London School of Hygiene & Tropical Medicine, London, UK; College of Health and Medical Sciences, Haramaya University, Harar, Ethiopia; College of Health and Medical Sciences, Haramaya University, Harar, Ethiopia; Department of Medical Microbiology, University of Ghana Medical School, University of Ghana, Accra, Ghana; Department of Medical Laboratory Sciences, School of Biomedical and Allied Health Sciences, University of Ghana, Accra, Ghana; KEMRI Centre for Geographic Medical Research, Kilifi, Kenya; Department of Medicine; Malawi-Liverpool Wellcome Programme, Kamuzu University of Health Sciences, Blantyre, Malawi; David Price Evans Infectious Diseases & Global Health Group, University of Liverpool, Liverpool, UK; Liverpool School of Tropical Medicine, Liverpool, UK; Department of Paediatrics and Child Health; Liverpool School of Tropical Medicine, Liverpool, UK; Department of Medicine; Department of Medical Microbiology, National Hospital Abuja, Abuja, Nigeria; Department of Medical Microbiology, National Hospital Abuja, Abuja, Nigeria; Nile University of Nigeria, Abuja, Nigeria; Department of Medical Microbiology, National Hospital Abuja, Abuja, Nigeria; Department of Paediatrics and Child Health; Kilimanjaro Clinical Research Institute, Kilimanjaro Christian Medical Centre, Moshi, Tanzania; Department of Medicine, University Teaching Hospital, Ministry of Health, Lusaka, Zambia; Centre for Infectious Disease Research in Zambia, Lusaka, Zambia; Infectious Disease Epidemiology Department, London School of Hygiene & Tropical Medicine, London, UK; Department of Medical Microbiology; Faculty of Medicine and Health Sciences, Stellenbosch University, Cape Town, South Africa; National Health Laboratory Service, Tygerberg Hospital, Cape Town, South Africa; College of Health and Medical Sciences, Haramaya University, Harar, Ethiopia; College of Health and Medical Sciences, Haramaya University, Harar, Ethiopia; Infectious Disease Epidemiology Department, London School of Hygiene & Tropical Medicine, London, UK; Department of Microbiology, Central Laboratory, Korle-Bu Teaching Hospital, Accra, Ghana; Department of Medical Microbiology, University of Ghana Medical School, University of Ghana, Accra, Ghana; Department of Public Health, Korle-Bu Teaching Hospital, Accra, Ghana; KEMRI Centre for Geographic Medical Research, Kilifi, Kenya; Department of Medicine, Malawi-Liverpool Wellcome Programme, Kamuzu University of Health Sciences, Blantyre, Malawi; Department of Medicine, Malawi-Liverpool Wellcome Programme, Kamuzu University of Health Sciences, Blantyre, Malawi; Liverpool School of Tropical Medicine, Liverpool, UK; Department of Medicine, Malawi-Liverpool Wellcome Programme, Kamuzu University of Health Sciences, Blantyre, Malawi; Department of Medical Microbiology, National Hospital Abuja, Abuja, Nigeria; Department of Pharmacy, National Hospital Abuja, Abuja, Nigeria; Department of Paediatrics and Child Health, Faculty of Medicine and Health Sciences, Stellenbosch University, Cape Town, South Africa; Kilimanjaro Clinical Research Institute-Kilimanjaro Christian Medical Centre, Moshi, Tanzania; Kilimanjaro Christian Medical University College, Moshi, Tanzania; Kilimanjaro Clinical Research Institute-Kilimanjaro Christian Medical Centre, Moshi, Tanzania; Pharmacy Department, University Teaching Hospital, Ministry of Health, Lusaka, Zambia; Centre for Infectious Disease Research in Zambia, Lusaka, Zambia; Pharmacy Department, University Teaching Hospital, Ministry of Health, Lusaka, Zambia; Department of Microbiology, University Teaching Hospital, Ministry of Health, Lusaka, Zambia

## Abstract

**Background:**

Bacteria of the order Enterobacterales are common pathogens causing bloodstream infections in sub-Saharan Africa and are frequently resistant to third-generation cephalosporin antibiotics. Although third-generation cephalosporin resistance is believed to lead to adverse outcomes, this relationship is difficult to quantify and has rarely been studied in this region. We aimed to measure the effects associated with resistance to third-generation cephalosporins in hospitalised patients with Enterobacterales bloodstream infection in Africa.

**Methods:**

We conducted a prospective, matched, parallel cohort study at eight hospitals across sub-Saharan Africa. We recruited consecutive patients of all age groups with laboratory-confirmed Enterobacterales bloodstream infection and matched them to at least one patient without bloodstream infection on the basis of age group, hospital ward, and admission date. Date of infection onset (and enrolment) was defined as the day of blood sample collection for culturing. Patients infected with bacteria with a cefotaxime minimum inhibitory concentration of 1 mg/L or lower were included in the third-generation cephalosporin-susceptible (3GC-S) cohort, and the remainder were included in the third-generation cephalosporin-resistant (3GC-R) cohort. The primary outcomes were in-hospital death and death within 30 days of enrolment. We used adjusted multivariable regression models to first compare patients with bloodstream infection against matched patients within the 3GC-S and 3GC-R cohorts, then compared estimates between cohorts.

**Findings:**

Between Nov 1, 2020, and Jan 31, 2022, we recruited 878 patients with Enterobacterales bloodstream infection (221 [25·2%] to the 3GC-S cohort and 657 [74·8%] to the 3GC-R cohort) and 1634 matched patients (420 [25·7%] and 1214 [74·3%], respectively). 502 (57·2%) bloodstream infections occurred in neonates and infants (age 0–364 days). *Klebsiella pneumoniae* (393 [44·8%] infections) and *Escherichia coli* (224 [25·5%] infections) were the most common Enterobacterales species identified. The proportion of patients who died in hospital was higher in patients with bloodstream infection than in matched controls in the 3GC-S cohort (62 [28·1%] of 221 *vs* 22 [5·2%] of 420; cause-specific hazard ratio 6·79 [95% CI 4·06–11·37] from Cox model) and the 3GC-R cohort (244 [37·1%] of 657 *vs* 115 [9·5%] of 1214; 5·01 [3·96–6·32]). The ratio of these cause-specific hazard ratios showed no significant difference in risk of in-hospital death in the 3GC-R cohort versus the 3GC-S cohort (0·74 [0·42–1·30]). The ratio of relative risk of death within 30 days (0·82 [95% CI 0·53–1·27]) also indicated no difference between the cohorts.

**Interpretation:**

Patients with bloodstream infections with Enterobacterales bacteria either resistant or susceptible to third-generation cephalosporins had increased mortality compared with uninfected matched patients, with no differential effect related to third-generation cephalosporin-resistance status. However, this finding does not account for time to appropriate antibiotic treatment, which remains clinically important to optimise. Measures to prevent transmission of Enterobacterales could reduce bloodstream infection-associated mortality from both drug-resistant and drug-susceptible bacterial strains in Africa.

**Funding:**

Bill & Melinda Gates Foundation.

## Introduction

Antimicrobial resistance (AMR) is a major global health challenge.^1^ A modelling study estimated that 4·95 million deaths associated with bacterial AMR occurred in 2019, including 1·27 million deaths attributable to bacterial AMR—these estimates were based on comparisons with hypothetical “no infection” and “susceptible infection” scenarios, respectively. Sub-Saharan Africa was identified as the region most intensely affected by AMR-attributable deaths, with bloodstream infections caused by drug-resistant *Klebsiella pneumoniae* and *Escherichia coli* (two species in the order Enterobacterales) being major contributors to this burden.^2^ Rates of resistance to third-generation cephalosporins are high in African countries, although few studies have measured the effects of this, or any, form of antibiotic resistance in this region.^3–6^

Measuring the effects of AMR is challenging because drug resistance occurs differentially by age, bacterial species, comorbidities, health-care exposure, and geographical location, all of which act as confounders in observational studies. Recent WHO recommendations on measuring the effects of AMR encourage the use of matched uninfected patients for comparison to better understand the effects of bloodstream infection.^7^

We aimed to measure the effects of third-generation cephalosporin resistance in bloodstream infections caused by Enterobacterales bacteria on mortality and length of hospital stay in sub-Saharan Africa. Our study was modelled on a previous European study of the impact of AMR^8,9^ and informed by a retrospective pilot study in six African hospitals^10^ and a single-hospital prospective pilot study in South Africa.^11^

## Methods

### Study design and participants

**Figure 1:**
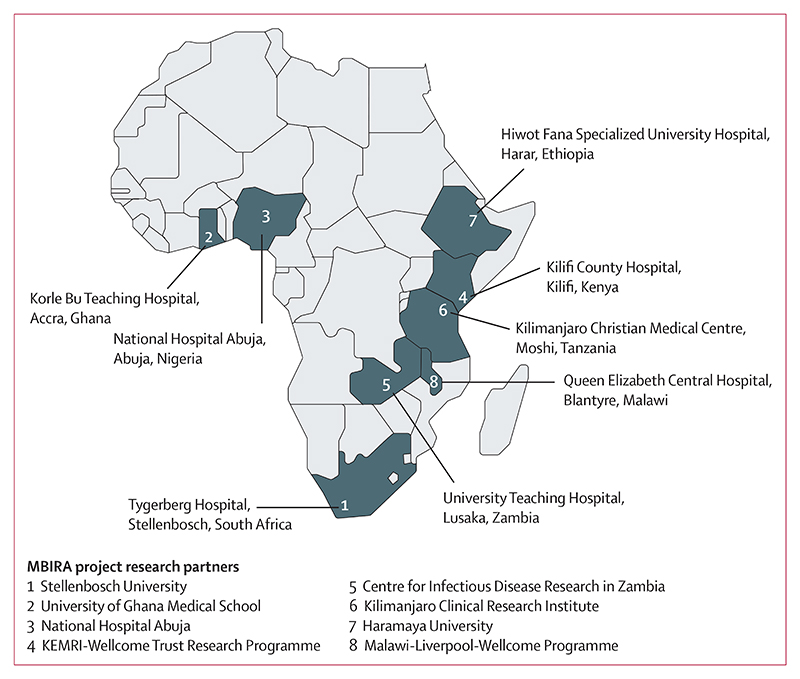
Participating study sites

The MBIRA study used a matched parallel cohort design for estimating AMR impact, with prospectively recruited patients with Enterobacterales bloodstream infection and individually matched patients without Enterobacterales bloodstream infection followed up in two cohorts: a third-generation cephalosporin-susceptible (3GC-S) cohort and a third-generation cephalosporin-resistant (3GC-R) cohort. This four-group design assumes that source populations for drug-resistant and drug-susceptible infections are different, precluding direct comparison. Eight public-sector hospitals in sub-Saharan Africa with well established microbiology services and relevant laboratory quality control measures participated in the study (figure 1). We previously described the characteristics of these hospitals, including blood culturing practices and access to antibiotic agents.^12^ One site, hospital 7 in Ethiopia, did not recruit adult patients, whereas all other study sites recruited across all age groups, classified as neonates (age 0–28 days), infants (age 29–364 days), children (age 1–14 years), and adults (age ≥15 years).

We attempted to recruit all consecutive inpatients with laboratory-confirmed Enterobacterales bloodstream infection during the study period. Patients who left hospital or died before blood culture results were available were recruited if appropriate consent could be obtained. We included cases of infection with any species within the order Enterobacterales, except for *Salmonella* species, which we considered to represent a distinct group for study purposes. Patients with a second pathogenic non-Enterobacterales isolate (eg, *Staphylococcus aureus*) in the same blood culture were ineligible for inclusion, although patients with multiple different Enterobacterales species in the same blood culture were eligible. Patients with bloodstream infection were excluded if no suitable matched patients were recruited, if their bacterial isolates did not reach the reference laboratory, or if there was a genus-level identification disagreement between the local and reference laboratories.

Ethical approval for this study was granted by the London School of Hygiene & Tropical Medicine ethics committee and relevant institutional bodies at all participating sites. Individual patients (or their relatives or guardians) gave written informed consent to participate in the study.

### Selection of matched uninfected patients

For each patient with bloodstream infection, uninfected patients were identified of the same age group in the same hospital ward and with admission date within 1 month before or after that of the infected patient. Patients were considered uninfected if they had no known bloodstream infection; thus, patients with other clinically suspected or laboratory confirmed forms of infection remained eligible for selection as matches. Adjacent age groups (eg, neonates and infants) were accepted if no suitable matches were available in the same age group. A final matching constraint (time-in-hospital criterion) was that the uninfected patients’ admissions were required to last at least as long (in days) as the interval from hospital admission to blood culture collection for the corresponding patient with bloodstream infection; this constraint was applied to avoid immortal time bias.^13^ We aimed to recruit two matched patients for each patient with bloodstream infection.

For each patient with bloodstream infection, we calculated a time-to-bloodstream infection interval as the number of days between their date of admission and date of blood culture. For each matched patient, their date of study enrolment was defined as their date of admission plus the time-to-bloodstream infection interval (in days) of the corresponding patient with bloodstream infection. If more than two uninfected patients were eligible for matching, researchers selected between them at random; our recommended method of randomisation was use of the Random: All Things Generator app, but we did not collect information on the approach used.

### Definitions and outcomes

For patients with bloodstream infection, we defined infection onset and date of enrolment as the date of the blood culture collection (day 0). Episodes of bloodstream infection with blood cultures collected on the first or second day of hospital admission were considered to be community-acquired; thereafter, bloodstream infection episodes were considered to be hospital-acquired. Bloodstream infection episodes occurring within 2 days of admission in patients with a history of hospitalisation in the preceding 30 days were considered to be health-care-associated bloodstream infections.

The primary study exposure was third-generation cephalosporin-resistance status, defined by cefotaxime minimum inhibitory concentration (MIC) from reference laboratory testing. Patients infected with bacteria with a cefotaxime MIC of 1 mg/L or lower were included in the 3GC-S cohort, while the remainder (including those with intermediate susceptibility status)^14^ were included in the 3GC-R cohort. Patients with polymicrobial bloodstream infections were included in the 3GC-R cohort if any infecting Enterobacterales species was resistant. The primary outcomes were in-hospital death and death within 30 days of enrolment.

### Data collection methods

At each site, clinical research staff recruited patients and collected clinical, laboratory, and outcome data, including making follow-up telephone calls to ascertain 30-day mortality outcomes.^15^ Data were obtained from medical records, patients, or attending physicians. Charlson Comorbidity Index scores were calculated and data were collected on HIV status, use of indwelling medical devices, and other relevant diseases. Data were entered into a REDCap database and checked for missing or inconsistent values. At final analysis, study subjects with unresolved missing or inconsistent key data were excluded from the analysis.

### Microbiological procedures

Staff at each hospital analysed blood cultures according to their current clinical and laboratory practices, as previously described.^12^ As preparation for the study, we provided training materials on good practices in blood culturing.^16,17^ We also monitored blood culture and contamination rates throughout the study. Bacterial isolates from enrolled patients with bloodstream infection episodes were stored locally until the end of the recruitment period and then transferred to the study reference laboratory at Stellenbosch University (Stellenbosch, South Africa). This laboratory is accredited by the South African National Accreditation System (ISO 15189). The VITEK 2 platform (bioMerieux, Marcy-l’Étoile, France) was used for species-level identification and antibiotic susceptibility testing, in accordance with published guidance.^14^ Microbiology results from the study reference laboratory were used for all analyses presented.

### Statistical analysis

We originally aimed to recruit 1200 patients with Enterobacterales bloodstream infection (exposed patients) across ten hospitals in sub-Saharan Africa, based on an expected odds ratio of 3·0 for 30-day mortality risk between the 3GC-R cohort and the 3GC-S cohort. By the start of the fieldwork period in late 2020, we had only been able to identify eight African hospitals that met suitable quality criteria and were willing to participate in the study. We therefore pragmatically reduced the planned number of hospitals and patients with bloodstream infection, maintaining a target of 120 exposed patients per hospital (960 exposed patients in total), as study timelines and budgets had already been agreed on this basis. This number was judged to be the practical upper limit of recruitment at most of the hospitals within the time and budget available, and was estimated to remain adequately powered to detect an effect of the size described above.

Analyses were done with Stata (version 17). We followed analytical approaches developed in our retrospective^10^ and prospective^11^ pilot studies. Before completing data collection, we wrote a statistical analysis plan.^15^ The impact of bloodstream infection on outcomes was evaluated in two steps. First, exposed patients were compared against matched, unexposed patients in regression models within each cohort (3GC-R and 3GC-S). These estimates were then used to generate a resistant-versus-susceptible ratio-of-ratios to assess the effect of resistance to third-generation cephalosporins.^18^ Robust standard errors were used to account for the matched design. For in-hospital outcomes, we grouped patients who were transferred or who absconded or had unknown outcome with those who were discharged alive.

To better understand aetiology, two cause-specific Cox regression models were used. One estimated the hazard ratios (HRs) of the effect of bloodstream infection on in-hospital mortality with censoring at the competing event of discharge, and the second examined the effect on risk of discharge with censoring at inpatient death.^19^ We generated cumulative mortality incidence curves for visualisation and examination of any violations of the proportional hazards assumption.^20^ To assess the effect on overall in-hospital mortality, considering the competing event of discharge, we used Fine and Gray extended Cox regression models.^21^ We used generalised linear models with Poisson distribution and log link to estimate the relative risk (RR) of 30-day mortality; patients with unknown 30-day outcomes were excluded from this analysis.^22^ Our multivariable models included variables we considered to be clinically plausible confounders representing the pre-existing health of individual patients (age, number of indwelling medical devices, HIV status, and Charlson Comorbidity Index). For patients with unknown HIV status, we used multiple imputation with chained equations to impute categorical HIV status.

We did not include variables representing acute disease severity measured at the time of infection (such as Pitt bacteraemia score or qSOFA score) in our models as we considered these variables to lie on the causal pathway between infection and death; in other words, we considered these variables to result from the infection, rather than cause it, and hence not appropriate to adjust for in these analyses. We planned subgroup analyses by bacterial species, site, age group, and location of onset for the in-hospital death outcome. We planned sensitivity analyses in which the following patient groups were excluded from analysis: early deaths and discharges (days 0–1); and patients who were transferred, absconded, or had unknown hospital outcomes. After initial analysis of data, we also conducted the following unplanned sensitivity analyses: excluding patients with bloodstream infection with MIC values between 2 mg/L and 32 mg/L; excluding data from entire sites, one by one; and using preterm birth as an additional adjustment term for the neonatal subgroup analysis. These further analyses all used only the cause-specific Cox regression model with the in-hospital death outcome.

### Role of the funding source

The funder of the study had no role in study design, data collection, data analysis, data interpretation, or writing of the report.

## Results

**Figure 2:**
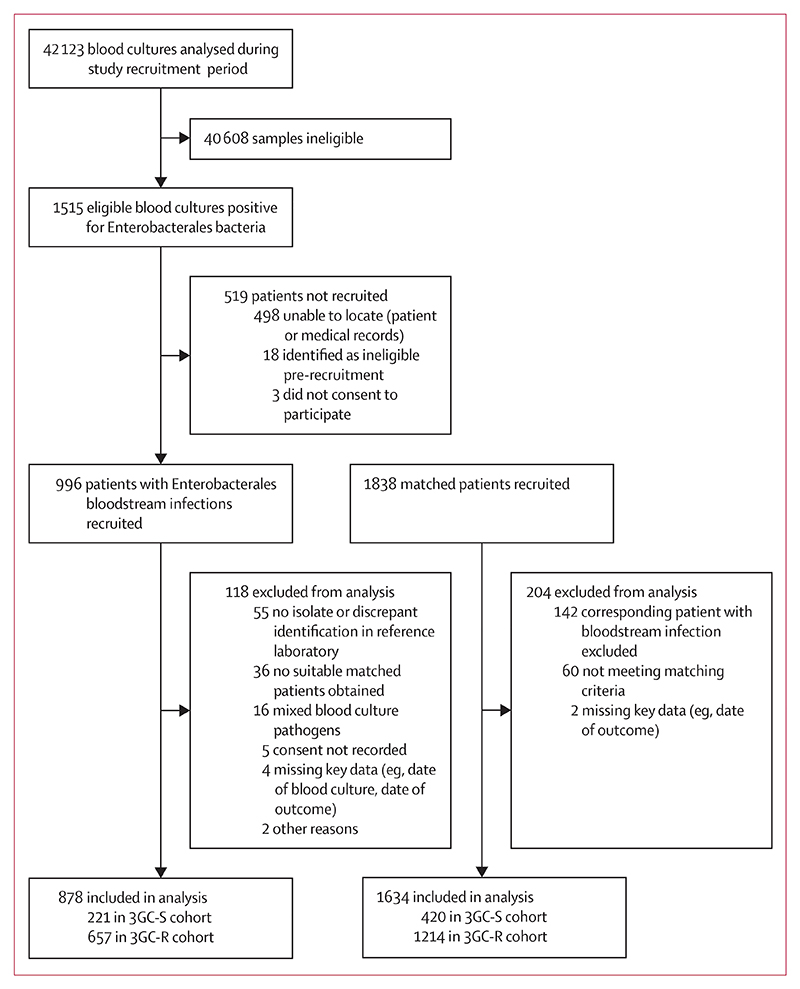
Study profile

Between Nov 1, 2020, and Jan 31, 2022, 1515 blood cultures with microbiologically confirmed Enterobacterales bacteraemia were identified across all participating hospitals (figure 2). For 519 patients, either the patient or medical records could not be located, the patient was ineligible for the study, or informed consent was not obtained. We recruited 996 patients with bloodstream infection and, after subsequent exclusions, 878 patients with bloodstream infection and 1634 matched patients remained in the analysis, with a mean of 1·9 matched patients per patient with bloodstream infection.

Of the included patients with bloodstream infection, 221 were included in the 3GC-S and 657 in the 3GC-R cohort, each with at least one matched patient (table 1). Across the two cohorts, age distributions were similar, with neonates being the most common age group. Among the 422 neonatal patients with bloodstream infection, 207 (49%) were born preterm (gestational age at birth <37 weeks).

In the 3GC-S cohort, 70 (31·7%) of 221 patients with bloodstream infection were recruited at hospital 1 (South Africa). In the 3GC-R cohort, hospital 7 (Ethiopia) contributed the most patients with bloodstream infection. Hospital 4 (Kenya) contributed the smallest number of patients with bloodstream infection to the study, equally distributed between the two cohorts. 132 (5·3%) of 2512 patients had known HIV-positive status (either on antiretroviral therapy or currently untreated), with a slightly higher proportion among patients with bloodstream infection in the 3GC-S cohort. The median Charlson Comorbidity Index score was 0 in all patient groups, with a higher 95th percentile score among patients with bloodstream infection than among matched patients in both cohorts (table 1).

For patients with bloodstream infection, the median interval from hospital admission to blood culture collection (enrolment) was 1 day (IQR 0–5) in the 3GC-S cohort and 2 days (0–7) in the 3GC-R cohort. This interval also varied between sites, being lowest (median 0 days) in the hospitals in Kenya and Ethiopia and highest in the hospitals in Zambia (5·5 days) and South Africa (7 days).

Among the 878 bloodstream infections included, monomicrobial infections caused by *K pneumoniae* (393 [44·8%]) and *E coli* (224 [25·5%]) were the most common (table 2). 46 patients had polymicrobial bloodstream infection (all with two Enterobacterales isolates), and were classified separately from monomicrobial infections. Among individual pathogens, 130 (58·0%) of 224 *E coli* and 350 (89·1%) of 393 *K pneumoniae* bloodstream infections were categorised as resistant to third-generation cephalosporins. In terms of onset, 3GC-S bloodstream infections were most commonly community-acquired (91 [41·2%] of 221), whereas 3GC-R infections were most frequently hospital-acquired (350 [53·3%] of 657).

The proportion of patients who died in hospital was higher among those with bloodstream infection than among matched patients in both the 3GC-S cohort (62 [28·1%] of 221 patients with bloodstream infection *vs* 22 [5·2%] of 420 matched patients) and 3GC-R cohort (244 [37·1%] of 657 *vs* 115 [9·5%] of 1214; table 1). For the hospital outcome, 297 (11·8%) of all 2512 patients were either transferred, absconded, or had unknown outcome. Similarly, for the 30-day mortality outcome, vital status (as ascertained by telephone calls) was unknown for 287 (11·4%) patients overall. Among matched patients, in-hospital mortality was substantially higher among those in the 3GC-R cohort (115 [9·5%] of 1214) than among those in the 3GC-S cohort (22 [5·2%] of 420), and a similar pattern was observed for the 30-day mortality outcome.

In our main analyses, we compared minimally adjusted (for age and study site only) and fully adjusted models to examine how much difference adjustment made to our estimates (table 3). Across all analyses, minimally and fully adjusted models produced very similar results; thus, we report based on fully adjusted models, but the same interpretations would be made from minimally adjusted models. We graphically examined cumulative incidence plots for violations of the proportional hazards assumption (appendix p 4). In event-specific Cox regression models, compared with matched uninfected patients, patients with bloodstream infection in the 3GC-S cohort had substantially increased instantaneous hazard of in-hospital death (cause-specific HR 6·79 [95% CI 4·06–11·37]) and a reduced rate of hospital discharge (0·72 [0·59–0·87]), indicating that these patients had prolonged hospital admissions. In the 3GC-R cohort, patients with bloodstream infection also had an increased instantaneous hazard of in-hospital death (cause-specific HR 5·01 [95% CI 3·96–6·32]) and a reduced hazard of hospital discharge (0·83 [0·75–0·92]). The overall ratios of cause-specific HRs in patients with bloodstream infection in the 3GC-R versus the 3GC-S cohort was 0·74 (95% CI 0·42–1·30) for in-hospital death and 1·16 (0·93–1·45) for hospital discharge; these confidence intervals (crossing 1·00) indicate no association between third-generation cephalosporin resistance and in-hospital death or discharge risk in these patients.

In the Fine and Gray models for in-hospital death, the subdistribution HRs, which indicate the effect on cumulative mortality over the entire admission, showed similar patterns to the event-specific Cox models. Estimates were slightly higher than the corresponding event-specific Cox models for death because the Fine and Gray models combine the effects of the Cox models for increased in-hospital death and prolonged admission. The ratio of subdistribution HRs for in-hospital death between the two cohorts also crossed 1·00 (0·75 [95% CI 0·42–1·32]), indicating no association of third-generation cephalosporin-resistance status with death rate. For 30-day death risk, we found that the ratio of effect estimates was also not significantly different (ratio of RR 0·82 [95% CI 0·53–1·27]), corroborating the findings of the in-hospital death outcome analyses.

**Figure 3:**
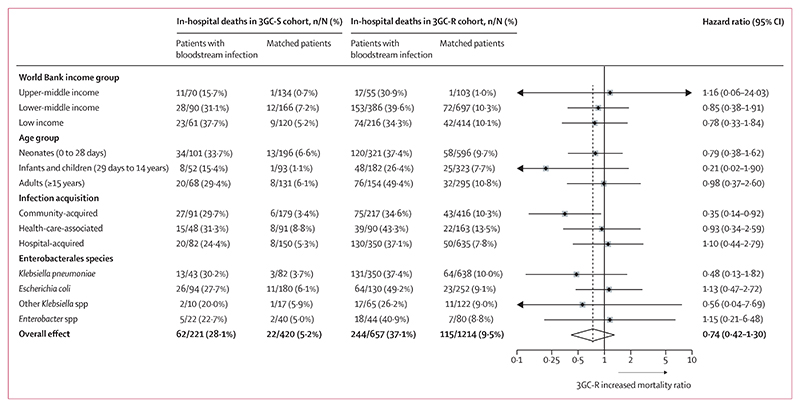
Cause-specific hazard ratios for in-hospital death in subgroups of 3GC-R cohort versus 3GC-S cohort 3GC-S=third-generation cephalosporin-susceptible. 3GC-R=third-generation cephalosporin-resistant.

In subgroup analyses (figure 3), we found no association of third-generation cephalosporin-resistance status with mortality when considering individual bacterial groups and age groups for the in-hospital death outcome. There were non-significant trends towards lower mortality associated with 3GC-R status for *K pneumoniae*, other *Klebsiella* species, and infants or children. For the two most common bacterial species, *K pneumoniae* and *E coli*, species-specific patient descriptive information, including outcome data, is given in the appendix (pp 2–3). We also examined effects grouped by World Bank income level (an unplanned additional analysis as some individual hospitals had non-calculable values), which all also showed no evidence of difference by subgroup. The only subgroup that clearly showed a distinct effect was community-acquired bloodstream infection; this subgroup was associated with a significantly reduced effect on mortality for 3GC-R infections compared with 3GC-S infections (cause-specific HR 0·35 [95% CI 0·14–0·92]). Health-care-associated or hospital-acquired bloodstream infections in the 3GC-R cohort showed no difference in mortality compared with the bloodstream infections in the 3GC-S cohort. We conducted various sensitivity analyses as described in the methods section. These analyses all reached similar conclusions for the in-hospital death outcome.

## Discussion

This is the largest prospective African study examining the effects of third-generation cephalosporin resistance in Enterobacterales bloodstream infection. It benefits from complete data and standardised microbiological testing at one laboratory in South Africa. We used individually matched patients without infection recruited from the same hospital wards to account for possible confounding, meaning that our final estimates were ratios-of-ratios between the 3GC-S and 3GC-R cohorts. This design choice is supported by wide differences in the prevalence of third-generation cephalosporin resistance by site and high frequencies of hospital-acquired infection. Some sites had very infrequent occurrences of 3GC-S bloodstream infection, which would have hindered direct comparison at the site level. Use of matched uninfected patients in AMR effect analyses is encouraged by WHO.^7^

In our central analysis, we found marked effects of bloodstream infection on risk of death and prolongation of hospital stay when compared with matched uninfected patients. However, when comparing outcomes between cohorts among patients with bloodstream infection, we found no apparent differences by third-generation cephalosporin susceptibility status for either death or length of hospital admission. Although these were unexpected results, they are qualitatively the same as those from our pilot studies.^10,11^ We found that neonates, especially those born preterm, were the most common age group affected by bloodstream infection in these African hospitals. About two-thirds of all patients with bloodstream infection had recent or current health-care exposure (ie, had onset classified as health-care-associated or hospital-acquired) and *K pneumoniae* was by far the most common species of Enterobacterales causing bloodstream infection in these hospitals, with a high level of third-generation cephalosporin resistance in this species (350 [89%] of 393 bloodstream infections across all sites). These patterns are consistent with other research on bloodstream infection in Africa.^4,23^ We also found higher rates of third-generation cephalosporin resistance in *E coli* infections (130 [58%] of 224 across all sites) than has historically been described in this region.^2,3^

Subgroup and sensitivity analyses showed that most subgroups had similar qualitative interpretations, although community-acquired bloodstream infection had a substantially lower death rate associated with 3GC-R status (cause-specific HR 0·35 [95% CI 0·14–0·92]). This finding implies that community-acquired bloodstream infection had an inverse relationship between third-generation cephalosporin resistance and risk of death, which was not present for nosocomial infections. Associations between bacterial virulence and resistance depend on multiple factors, including level of antibiotic use in the local environment;^24^ thus, differences in virulence–AMR associations in different locations of acquisition are a possible explanation for this finding.

Our study design used separate comparison groups for patients infected with Enterobacterales species resistant or susceptible to third-generation cephalosporins, first calculating effects separately based on a counterfactual of no infection. Estimates were then combined to give a ratio-of-ratios describing the effect of resistance over susceptibility, incorporating the underlying differences between patient groups. This approach is different to the direct comparisons between susceptible and resistant infections typically used for AMR impact analyses. This design acknowledges different characteristics between patients with drug-resistant and drug-susceptible infections, representing a high-quality method for minimising confounding, well suited to this study. This design was selected at the start of the project and followed through two pilot studies to this planned main analysis. Post hoc, differences observed between the two matched uninfected groups further support this design choice.

We sought possible reasons for our unexpected results. Chance findings can never be ruled out, but this large study had complete data and found consistent results across pilot studies, subgroups, and sensitivity analyses. As such, another study following this design would be unlikely to have reached a different conclusion. This study did not meet its originally planned sample size, mainly due to difficulty in identifying suitable participating sites.

We judged our study to be at low risk of selection bias as similar approaches to recruit patients with bloodstream infection were used at all sites, and we achieved good rates of follow-up. However, several of the participating hospitals had blood culture testing done for a low proportion of patients,^12^ which can lead to late and unrepresentative identification of infections.^25^ This could have resulted in ascertainment bias in this study, especially if a greater proportion of 3GC-S infections were missed. Unfortunately, greater financial support to improve blood culturing rates at all sites was not possible within our study budget. Widespread use of antibiotics before hospital admission or between admission and blood culturing could have impeded identification of 3GC-S bloodstream infections. We judged that the use of antibiotics between admission and blood culturing might have led to some under-identification of 3GC-S bloodstream infections, but probably not in sufficient numbers to change the qualitative findings of the study.

Any AMR impact analysis should attempt to control for patient-level and hospital-level confounding, such that differences in pathogen phenotype alone are measured. Appropriately controlling for such confounding is difficult, as these concepts are fundamentally difficult to measure, and residual confounding therefore cannot be ruled out. In particular, the Charlson Comorbidity Index is poorly suited for classifying comorbidities in neonates, but there is no alternative index suitable across all age groups.

Any unexpected finding could represent a true result, meaning that an existing paradigm needs to be updated. Adverse effects associated with AMR are typically thought to be realised through one of three different patient-level pathways: higher frequency of inappropriate antibiotic treatment (ie, treatment non-concordant with in vitro susceptibility testing), reduced effectiveness of second-line antibiotic treatment (eg, vancomycin *vs* flucloxacillin), or increased virulence of resistant pathogens. This analysis does not describe initial or overall antibiotic therapy or examine how it influences patient outcomes. We previously described the within-hospital availability of various antibiotic agents in these hospitals and broad patterns of antibiotic use in patients with bloodstream infection.^12^ Broadly speaking, in most of these hospitals, either meropenem or amikacin were available for use in patients with bloodstream infection. Use of such antibiotic treatments might provide some explanation for our study findings, but relating time to appropriate antibiotic treatment to patient outcomes^26^ is complex and beyond the scope of this analysis. We plan to investigate whether the study isolates show a relationship between carriage of resistance and virulence genes,^27^ which could provide an explanation for some of our subgroup analysis findings. Limitations in health care for African populations, including late presentation to services and scarce facilities for intensive care, could also mean that third-generation cephalosporin-resistance status is obscured as a factor contributing to bloodstream infection-associated mortality in this region and, hence, does not appear as an association. This explanation is supported by the high in-hospital mortality for patients with bloodstream infection in this study (306 [34·9%] of 878), which is much higher than that in the European study^9^ on which it was modelled (229 [18·9%] of 1210).

Comparing these results with other research, our retrospective pilot study^10^ using the same design with a large historical dataset (albeit mainly from two hospitals in Kenya and South Africa) also found no difference in mortality between 3GC-R and 3GC-S types of *E coli* and *K pneumoniae* bloodstream infection. A recent single-hospital study of the effects of third-generation cephalosporin from Queen Elizabeth Central Hospital in Malawi (hospital 8 in this study), which has very high blood culture rates, recently found an effect on mortality of third-generation cephalosporin-resistance status in adjusted analyses. Although that study did not include uninfected patients for comparison, it did have additional resources to recruit or identify all individuals with bloodstream infection.^28^ Other African studies in this subject area are rare.^5,29^ Among studies examining this question in Africa, estimates of the effect of third-generation cephalosporin resistance on mortality thus appear inconsistent and related to methodology. In research outside of Africa, some other studies have also not found a difference in mortality between infections with bacteria resistant or susceptible to third-generation cephalosporins,^30,31^ but contextual factors differ.

Modelling of the effects of AMR on mortality internationally is difficult and requires many estimates, often based on scarce primary data. The effects of AMR can be evaluated under two scenarios: a replacement scenario, wherein resistant infections occur in place of equivalent susceptible infections, as measured by a ratio-of-ratios in this study; and an addition scenario, wherein resistant infections occur in place of no infection, equivalent to the within-cohort estimates in this study. Although the replacement scenario is more widely used, a recent discussion considered which was more relevant on a case-by-case basis.^32^ Interventions relating to reducing pathogen transmission (including in-hospital infection control measures and vaccination) and improving sepsis care^33^ would potentially reduce both resistant and susceptible infections—the preventable burden is then best estimated by the addition scenario. A major modelling study used both comparison scenarios to give upper and lower bounds of global AMR impacts.^2^ In that study, an estimated relative risk of mortality of 1·37 for third-generation cephalosporin resistance in *E coli* and 1·36 in *K pneumoniae* was used. These figures were applied globally, including all ages and infection types. As our mortality effect estimates for third-generation cephalosporin resistance differ substantially from these figures and as *E coli* and *K pneumoniae* were ranked among the top regional pathogens, African AMR impact estimates might require re-evaluation.

This large prospective African study that included the use of uninfected matched patients found substantial mortality associated with Enterobacterales bloodstream infection. Although our study is not without limitations, we found no evidence of a differential effect between third-generation cephalosporin-resistant and third-generation cephalosporin-susceptible forms of infection. Corroboration of these findings is needed, but these results imply that the most useful regional priorities for reducing bloodstream infection-associated mortality in sub-Saharan Africa would be controlling in-hospital transmission of Enterobacterales and improving care for patients with acute illness. Implementing such strategies should reduce the effects of both drug-resistant and drug-susceptible Enterobacterales bloodstream infections.

## Supplementary Material

supplementary materials

## Figures and Tables

**Table 1:** Profile of bloodstream infection patients and matched patients

	3GC-S cohort			3GC-R cohort	
	3GC-S bloodstream infection (n=221)	Matched uninfected patients (n=420)		3GC-R bloodstream infection (n=657)	Matched uninfected patients (n=1214)
**Patient characteristics**					
Age group					
Neonates (0−28 days)	101 (45·7%)	196 (46·7%)		321 (48·9%)	596 (49·1%)
Infants (29·364 days)	18 (8.1%)	27 (6·4%)		62 (9·4%)	104 (8·6%)
Children (1·14 years)	34 (15·4%)	66 (15·7%)		120 (18·3%)	219 (18·0%)
Adults (≥15 years)	68 (30·8%)	131 (31·2%)		154 (23·4%)	295 (24·3%)
Preterm birth* in neonatal group	42/101 (41·6%)	89/196 (45·4%)		169/321 (52·6%)	287/596 (48·2%)
Sex					
Female	95 (43·0%)	192 (45·7%)		295 (44·9%)	578 (47·6%)
Male	126 (57·0%)	228 (54·3%)		362 (55·1)	636 (52·4%)
Recruitment site					
Hospital 1, South Africa	70 (31·7%)	134 (31·9%)		55 (8·4%)	103 (8·5%)
Hospital 2, Ghana	37 (16·7%)	71 (16·9%)		88 (13·4%)	167 (13·8%)
Hospital 3, Nigeria	18 (8.1%)	27 (6·4%)		85 (12·9%)	140 (11·5%)
Hospital 4, Kenya	10 (4·5%)	21 (5·0%)		11 (1·7%)	32 (2·6%)
Hospital 5, Zambia	10 (4·5%)	19 (4·5%)		102 (15·5%)	199 (16·4%)
Hospital 6, Tanzania	15 (6·8%)	28 (6·7%)		100 (15·2%)	159 (13·1%)
Hospital 7, Ethiopia	30 (13·6%)	58 (13·8%)		137 (20·9%)	256 (21·1%)
Hospital 8, Malawi	31 (14·0%)	62 (14·8%)		79 (12·0%)	158 (13·0%)
HIV status					
Negative	141 (63·8%)	274 (65·2%)		389 (59·2%)	734 (60·5%)
Positive, on ART	15 (6·8%)	21 (5·0%)		30 (4·6%)	56 (4·6%)
Positive, not on ART	1 (0·5%)	2 (0·5%)		1 (0·2%)	6 (0·5%)
Unknown, including exposed children	64 (29·0%)	123 (29·3%)		237 (36·1%)	418 (34·4%)
Median Charlson Comorbidity Index score	0 (6)†	0 (3) †		0 (5) †	0 (2) †
Median number of indwelling medical devices	1 (1·2)	1 (1·1)		1 (1–2)	1 (1–1)
Median time from admission to enrolment, days	1 (0–5)	1 (0–5)		2 (0–7)	2 (0–7)
**Outcomes**					
Hospital outcome					
Discharged	136 (61·5%)	327 (77·9%)		348 (53·0%)	961 (79·2%)
Died	62 (28·1%)	22 (5·2%)		244 (37·1%)	115 (9·5%)
Transferred	14 (6·3%)	45 (10·7%)		12 (1·8%)	44 (3·6%)
Absconded or unknown	9 (4·1%)	26 (6·2%)		53 (8·1%)	94 (7·7%)
Median length of stay after enrolment, days	9 (4–18)	10 (6–17)		7 (3–14)	9(5–19)
Person-days of in-hospital observation	3483	5569		7479	16771
Crude rate of in-hospital deaths per 1000 person-days (95% CI)	17·80 (13·88–22·83) ‡	3·95 (2·60–6·00) ‡		32·62 (28·78–36·99) ‡	6·86 (5·71–8·23) ‡
30-day outcome					
Alive	144 (65·2%)	356 (84·8%)		324 (49·3%)	901 (74·2%)
Died	65 (29·4%)	30 (7·1%)		260 (39·6%)	145 (11·9%)
Unknown	12 (5·4%)	34 (8·1%)		73 (11·1%)	168 (13·8%)

Data are n (%), n/N (%), or median (IQR), except where otherwise specified. 3GC-S=third-generation cephalosporin-susceptible. 3GC-R=third-generation cephalosporin-resistant. ART=antiretroviral therapy.

* Defined as birth at <37 weeks’ gestation. †Values in parentheses are 95th percentiles; IQRs are not presented as values were all 0. ‡Values in parentheses are 95% CIs.

**Table 2:** Characteristics of Enterobacterales bloodstream infections

	3GC-S cohort (n=221)	3GC-R cohort (n=657)
**Bacterial species**		
*Escherichia coli*	94 (42·5%)	130 (19·8%)
*Klebsiella pneumoniae*	43 (19·5%)	350 (53·3%)
Other *Klebsiella* spp	10 (4·5%)	65 (9·9%)
*Enterobacter* spp	22 (10·0%)	44 (6·7%)
*Serratia* spp	29 (13·1%)	8 (1·2%)
*Pantoea* spp	4 (1·8%)	21 (3·2%)
Other Enterobacterales spp	9 (4·1%)	3 (0·5%)
Polymicrobial*	10 (4·5%)	36 (5·5%)
**Onset location**		
Community-acquired	91 (41·2%)	217 (33·0%)
Health-care-associated	48 (21·7%)	90 (13·7%)
Hospital-acquired	82 (37·1%)	350 (53·3%)
**Presumed source of bloodstream infection**		
Bone or joint	0	5 (0·8%)
CNS focus	11 (5·0%)	30 (4·6%)
Post-intervention	3 (1·4%)	6 (0·9%)
Ear, nose, or throat	0	5 (0·8%)
Intra-abdominal	46 (20·8%)	94 (14·3%)
Intravascular	9 (4·1%)	43 (6·5%)
Lower respiratory tract	24 (10·9%)	70 (10·7%)
Skin or soft tissue	8 (3·6%)	10 (1·5%)
Genitourinary	27 (12·2%)	48 (7·3%)
Maternal infection (neonates)	9 (4·1%)	16 (2·4%)
Unknown	84 (38·0%)	330 (50·2%)

3GC-S=third-generation cephalosporin-susceptible. 3GC-R=third-generation cephalosporin-resistant.

* Any two different Enterobacterales spp isolates in same blood culture.

**Table 3:** Effect of third-generation cephalosporin resistance in Enterobacterales bloodstream infections

	In-hospital outcomes			Death within 30 days: relative risk
	Death: cause-specific HR*	Discharge: cause-specific HR*	Death: sub-distribution HR †	
**on *vs* matched patients**				
Minimally adjusted	6·64 (4·14–10·64)	0·68 (0·56–0·82)	6·90 (4·23–11·25)	3·98 (2·70–5·89)
Fully adjusted	6·79 (4·06–11·37)	0·72 (0·59–0·87)	6·84 (4·06–11·52)	3·74 (2·50–5·59)
**3GC-R cohort: patients with bloodstream infection *vs* matched patients**				
Minimally adjusted	4·83 (3·88–6·03)	0·82 (0·74–0·91)	5·02 (4·04–6·26)	3·23 (2·71–3·84)
Fully adjusted	5·01 (3·96–6·32)	0·83 (0·75–0·92)	5·11 (4·07–6·43)	3·06 (2·56–3·65)
**3GC-R cohort *vs* 3GC-S cohort**				
Minimally adjusted	0·73 (0·43–1·23)	1·21 (0·98–1·51)	0·73 (0·43–1·24)	0·81 (0·53–1·24)
Fully adjusted	0·74 (0·42–1·30)	1·16 (0·93–1·45)	0·75 (0·42–1·32)	0·82 (0·53–1·27)

Values in parentheses are 95% CIs. In-hospital outcome models use the full analysis dataset (n=2512). 30-day mortality models include only patients with a known 30-day outcome (n=2225). All models include study site, either as strata (Cox regression models) or as fixed effects (other models). Minimally adjusted models are adjusted for age category only. Fully adjusted models are adjusted for age category, HIV status (including multiple imputation where HIV status is unknown), number of indwelling devices, and categorical Charlson Comorbidity Index. 3GC-S=third-generation cephalosporin-susceptible. 3GC-R=third-generation cephalosporin-resistant. HR=hazard ratio.

* Cox regression model.

† Fine and Gray extended Cox regression model.

## Data Availability

Datasets for this paper are available on request through the London School of Hygiene & Tropical Medicine Data Compass site at https://doi.org/10.17037/DATA.00003168. These data include cleaned de-identified participant data, data dictionary, and datasets relating to human participants, antibiotic usage, bacterial isolates, and hospital characteristics. Additional documents available include a statistical analysis plan, specimen informed consent form, and study manual. These data will be available on request to any scientific researcher, without further restriction. These data will be available once appropriate permission has been obtained for anonymous data sharing from the London School of Hygiene & Tropical Medicine Ethics Committee.

## Research in context

### Evidence before this study

Third-generation cephalosporin resistance is common in Enterobacterales bacterial species, such as *Escherichia coli* and *Klebsiella pneumoniae* in African countries, as recently modelled in the GRAM study. However, data from Africa linking drug resistance to clinical outcomes are extremely sparse. A 2020 systematic review by Lester and colleagues of the prevalence and outcomes of this form of drug resistance in sub-Saharan Africa found very few studies examining this issue, stating, “The striking lack of mortality data … is therefore a major barrier to a comprehensive understanding of the burden of AMR [antimicrobial resistance] in this setting.”

### Added value of this study

We provide results from a large, prospective study in eight sub-Saharan African hospitals, providing empirical data on the effect of AMR in bloodstream infections in this region. We found that patients with Enterobacterales bloodstream infections had substantially higher mortality compared with individually matched uninfected patients. Unexpectedly, we did not identify an additional effect of third-generation cephalosporin resistance on outcomes of Enterobacterales bloodstream infection in this setting.

### Implications of all the available evidence

Further work is needed to corroborate the finding of an absence of excess mortality caused by this important form of AMR in African hospital settings. These data indicate that bloodstream infections impose an important health burden in sub-Saharan African hospitals, and many of these infections appear to be acquired in hospital. Interventions to reduce the impact of these infections in Africa should concentrate on preventing in-hospital transmission of pathogens, promoting the development of relevant vaccines, and enhancing the quality of care for patients with sepsis, which should all reduce the burden of both drug-resistant and drug-susceptible infections.
