# Supplementary material for: Mortality associated with third-generation cephalosporin resistance in Enterobacterales bloodstream infections at eight sub-Saharan African hospitals (MBIRA): a prospective cohort study
Source: Lancet Infect Dis. Author manuscript; Available in PMC 2024 Dec 9. (PMC7617135; doi:10.1016/S1473-3099(23)00233-5)
Supplement: supplementary materials [file EMS194772-supplement-supplementary_materials.pdf]

THE LANCET
Infectious Diseases

Supplementary appendix

This appendix formed part of the original submission and has been peer reviewed. We post it as supplied by the authors.

Supplement to: Aiken AM, Rehman AM, de Kraker MEA, et al. Mortality associated with third-generation cephalosporin resistance in Enterobacterales bloodstream infections at eight sub-Saharan African hospitals (MBIRA): a prospective cohort study. *Lancet Infect Dis* 2023; published online July 13. https://doi.org/10.1016/S1473-3099(23)00233-5.

Contents

Supplementary Table 1a: profile of E. coli BSI patients and matched patients only 2

Supplementary Table 1b: profile of K. pneumoniae BSI patients and matched patients only 3

Supplmentary Figure 1: Graphical representation of survival in-hospital from enrollment for different 3GC cohorts, A = 3GC-susceptible cohort, B = 3GC-resistant cohort 4

MBIRA study collaborators 5

Supplementary Table 1a: profile of E. coli BSI patients and matched patients only

	3GC-S cohort		3GC-R cohort	
	E. coli **3GC-S BSI (n=94)**	**Matched uninfected patients (n=180)**	***E. coli*** **3GC-R BSI (n=130)**	**Matched uninfected patients (n=252)**
**Individual characteristics**				
Age group (n, %)				
Neonates (0-28 days)	39 (41.5)	75 (41.7)	18 (13.8)	41 (16.3)
Infants (29 days-364 days)	7 (7.4)	12 (6.7)	11 (8.5)	22 (8.7)
Children (1-14 years)	11 (11.7)	21 (11.7)	18 (13.8)	29 (11.5)
Adults (> 14 years)	37 (39.4)	72 (40)	83 (63.8)	160 (63.5)
For neonatal age group only, Prematurity (<37/40) at birth, (%)	12/39 (30.8)	29/75 (38.7)	9/18 (50.0)	20/41 (48.8)
Female sex (n, %)	40 (42.6)	87 (48.3)	60 (46.2)	120 (47.6)
Site recruited (n, %)				
Hospital 1, South Africa	32 (34.0)	61 (33.9)	9 (6.9)	17 (6.7)
Hospital 2, Ghana	14 (14.9)	14 (14.9)	26 (20.0)	48 (19.0)
Hospital 3, Nigeria	3 (3.2)	4 (2.2)	8 (6.2)	15 (6.0)
Hospital 4, Kenya	9 (9.6)	19 (10.6)	7 (5.4)	22 (8.7)
Hospital 5, Zambia	2 (2.1)	4 (2.2)	42 (32.3)	82 (32.5)
Hospital 6, Tanzania	8 (8.5)	14 (7.8)	11 (8.5)	15 (6.0)
Hospital 7, Ethiopia	6 (6.4)	11 (6.1)	4 (3.1)	8 (3.2)
Hospital 8, Malawi	20 (21.3)	40 (22.2)	23 (17.7)	45 (17.9)
HIV status (n, %)				
Negative	66 (70.2)	128 (71.1)	77 (59.2)	159 (63.1)
Positive, on ART	10 (10.6)	14 (7.8)	18 (13.8)	25 (9.9)
Positive, not on ART	1 (1.1)	0 (0.0)	0 (0.0)	3 (1.2)
Unknown (inc. exposed children)	17 (18.1)	38 (21.1)	35 (26.9)	65 (25.8)
Charlson Comorbidity Index score: (median, 95th percentile)	0, 12	0, 3	0, 8	0, 6
Number of indwelling medical devices: (median, IQR)	1 (1, 2)	1 (1, 1)	1 (1, 2)	1 (0, 1)
Admission to enrolment, days: (median, IQR)	0 (0, 1)	0 (0, 1)	4 (0, 11)	3 (0, 10)
**Outcomes**				
Hospital outcome (n, %)				
Discharged	61 (64.9)	150 (83.3)	60 (46.2)	208 (82.5)
Died	26 (27.7)	11 (6.1)	64 (49.2)	23 (9.1)
Transferred	6 (6.4)	12 (6.7)	2 (1.5)	2 (0.8)
Absconded / unknown	1 (1.1)	7 (3.9)	4 (3.1)	19 (7.5)
Length of stay after enrolment, days: (median, IQR)	7 (4, 16)	10 (6, 17)	7 (2, 16)	13 (7, 23)
Person-days of in-hospital observation	1,089	2,425	1,460	2,425
Crude rate of in-hospital deaths (deaths/1,000 days, 95%CI)	23.89 (16.26-35.1)	4.54 (2.51-8.19)	43.84 (34.3-56.0)	5.65 (3.75-8.50)
30-day outcome (n, %)				
Alive	64 (68.1)	154 (85.6)	56 (43.1)	200 (79.4)
Died	28 (29.8)	12 (6.7)	66 (50.8)	28 (11.1)
Unknown	2 (2.1)	14 (7.8)	8 (6.2)	24 (9.5)

Supplementary Table 1b: profile of K. pneumoniae BSI patients and matched patients only

	3GC-S cohort		3GC-R cohort	
	***K. pneumoniae* in 3GC-S BSI (n=43)**	**Matched uninfected patients (n=82)**	***K. pneumoniae* in 3GC-R BSI (n=350)**	**Matched uninfected patients (n=638)**
**Individual characteristics**				
Age group (n, %)				
Neonates (0-28 days)	24 (55.8)	47 (57.3)	241 (68.9)	434 (68.0)
Infants (29 days-364 days)	3 (7.0)	6 (7.3)	21 (6.0)	38 (6.0)
Children (1-14 years)	6 (14.0)	9 (11.0)	33 (9.4)	63 (9.9)
Adults (> 14 years)	10 (23.3)	20 (24.4)	55 (15.7)	103 (16.1)
For neonatal age group only, Prematurity (<37/40) at birth, (%)	9/24 (37.5)	22/47 (46.8)	131/241 (54.4)	216 (49.8)
Female sex (n, %)	22 (51.2)	39 (47.6)	161 (46.0)	310 (48.6)
Site recruited (n, %)				
Hospital 1, South Africa	14 (32.6)	27 (32.9)	35 (10.0)	65 (10.2)
Hospital 2, Ghana	8 (18.6)	15 (18.3)	48 (13.7)	92 (14.4)
Hospital 3, Nigeria	3 (7.0)	4 (4.9)	35 (10.0)	57 (8.9)
Hospital 4, Kenya	0 (0.0)	0 (0.0)	3 (0.9)	8 (1.3)
Hospital 5, Zambia	2 (4.7)	4 (4.9)	51 (14.6)	99 (15.5)
Hospital 6, Tanzania	6 (14.0)	12 (14.6)	88 (25.1)	143 (22.4)
Hospital 7, Ethiopia	5 (11.6)	10 (12.2)	46 (13.1)	85 (13.3)
Hospital 8, Malawi	5 (11.6)	10 (12.2)	44 (12.6)	89 (13.9)
HIV status (n, %)				
Negative	24 (55.8)	48 (58.5)	238 (68.0)	437 (68.5)
Positive, on ART	2 (4.7)	5 (6.1)	8 (2.3)	24 (3.8)
Positive, not on ART	0 (0.0)	1 (1.2)	1 (0.3)	3 (0.5)
Unknown (inc. exposed children)	17 (39.5)	28 (34.1)	103 (29.4)	174 (27.3)
Charlson Comorbidity Index score: (median, 95th percentile)	0, 2	0, 2	0, 3	0, 2
Number of indwelling medical devices: (median, IQR)	1 (1, 2)	1 (1, 1)	1 (1, 2)	1 (1, 1)
Admission to enrolment, days: (median, IQR)	2 (0, 7)	1 (0, 7)	2 (0, 7)	2 (0, 7)
**Outcomes**				
Hospital outcome (n, %)				
Discharged	28 (65.1)	70 (85.4)	195 (55.7)	503 (78.8)
Died	13 (30.2)	3 (3.7)	131 (37.4)	64 (10.0)
Transferred	1 (2.3)	8 (9.8)	6 (1.7)	32 (5.0)
Absconded / unknown	1 (2.3)	1 (1.2)	18 (5.1)	39 (6.1)
Length of stay after enrolment, days: (median, IQR)	10 (5, 16)	10 (5, 15)	8 (3, 16)	9 (5, 19)
Person-days of in-hospital observation	643	1,025	4,399	9,021
Crude rate of in-hospital deaths (deaths/1,000 days, 95%CI)	20.22 (11.74-34.82)	2.93 (0.94-9.08)	29.78 (25.1-35.3)	7.09 (5.55-9.06)
30-day outcome (n, %)				
Alive	25 (58.1)	70 (85.4)	174 (49.7)	479 (75.1)
Died	14 (32.6)	6 (7.3)	137 (39.1)	75 (11.8)
Unknown	4 (9.3)	6 (7.3)	39 (11.1)	84 (13.2)

Supplmentary Figure 1: Graphical representation of survival in-hospital from enrollment for different 3GC cohorts, A = 3GC-susceptible cohort, B = 3GC-resistant cohort

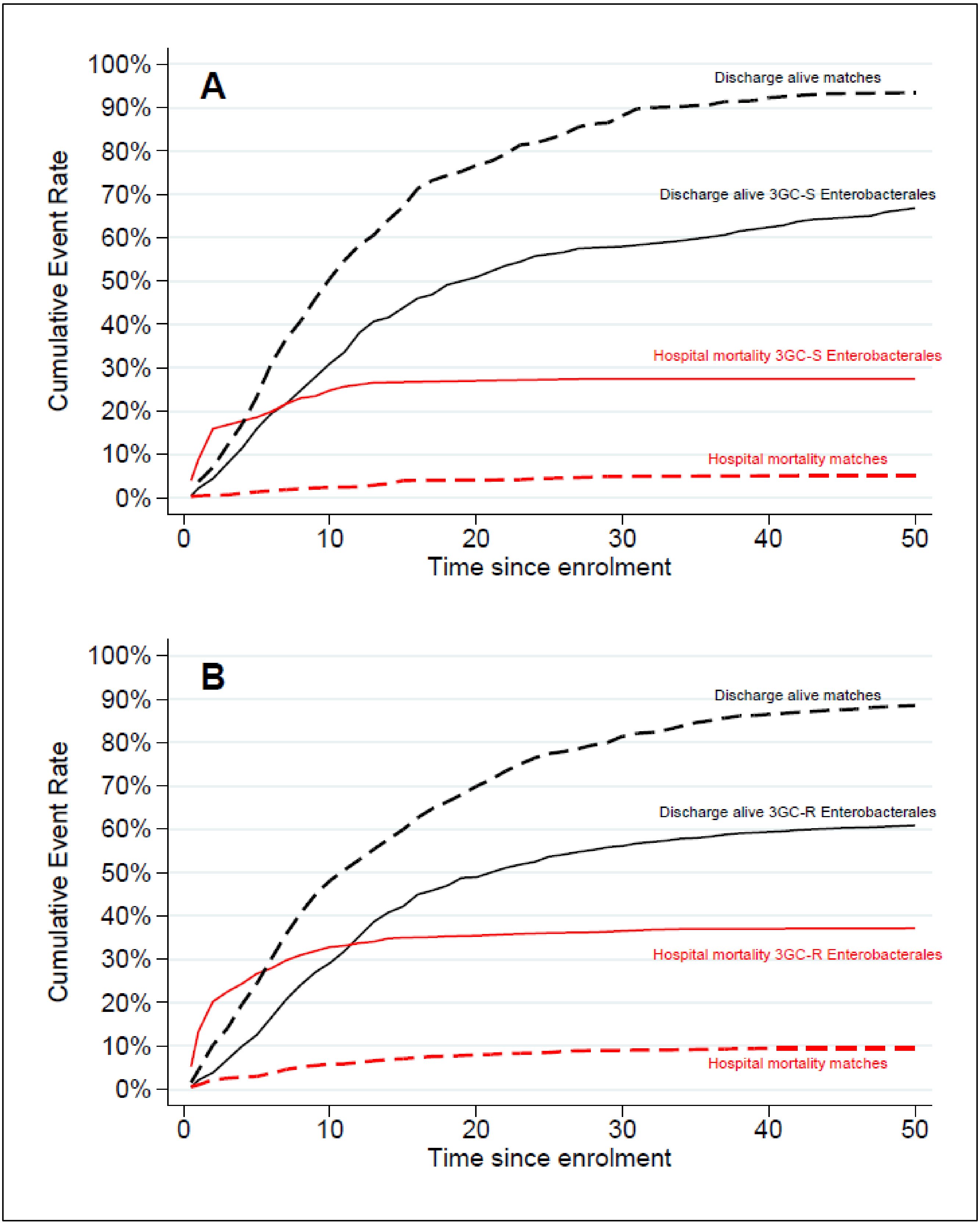

MBIRA study collaborators

Jabir Aliye, MD [3], Nega Assefa, PhD [3], Dumessa Edessa MSc [3], Joseph Oundo, PhD [1, 3], Mulu Berihun, MSc [3], Thomas Dankwah, MPhil [17], Mary M Osei, MPhil [4], Maude Fandoh, MPH [18], Margaret Gakpo, MSc [19], Caroline Mulunda, BSc [6], Benedict Mvera, BSc [6], Mabvuto Chimenya, BSc [7], Prof Nicholas Feasey, PhD [7,8], Jane Mallewa MD [7], Khadija Abdulraheem, MBBS [10], Tobechi A Akujobi, MBBS [10], Chinelo H Okonkwo, MPharm [20], Luzell Britz MSc [16], André N. H. Bulabula PhD [16], Aaqilah Fataar MBChB [12], Blandina T Mmbaga MD [13, 21], Neema Ng'unda MD [13], Uchizi Chirwa MSc [22], Nyambe Kakula BSc [15], Charles Muntemba MMed [22], Ruth Nakazawe MSc [23]

Affiliations

1 Infectious Disease Epidemiology Department, London School of Hygiene and Tropical Medicine, London, UK
2 Infection Control Program and WHO Collaborating Center on Patient Safety and Antimicrobial Resistance, University of Geneva Hospitals and Faculty of Medicine, Geneva, Switzerland
3 College of Health and Medical Sciences, Haramaya University, Harar, Ethiopia
4 Department of Medical Microbiology, University of Ghana Medical School, University of Ghana, Accra, Ghana
5 Department of Medical Laboratory Sciences, School of Biomedical and Allied Health Sciences, University of Ghana, Accra, Ghana
6 KEMRI Centre for Geographic Medical Research, Kilifi, Kenya
7 Department of Medicine, Malawi-Liverpool Wellcome Programme, Kamuzu University of Health Sciences, Blantyre, Malawi
8 Liverpool School of Tropical Medicine, Liverpool, United Kingdom
9 Department of Paediatrics and Child Health, Malawi-Liverpool Wellcome Programme, Kamuzu University of Health Sciences, Blantyre, Malawi
10 Department of Medical Microbiology, National Hospital Abuja, Abuja, Nigeria
11 Nile University of Nigeria, Abuja, Nigeria
12 Department of Paediatrics and Child Health, Faculty of Medicine and Health Sciences, Stellenbosch University, Cape Town, South Africa
13 Kilimanjaro Clinical Research Institute-Kilimanjaro Christian Medical Centre, Moshi, Tanzania
14 Department of Medicine, University Teaching Hospital, Ministry of Health, Zambia
15 Centre for Infectious Disease Research in Zambia, Lusaka, Zambia
16 Department of Medical Microbiology, Faculty of Medicine and Health Sciences, Stellenbosch University, Cape Town, South Africa
17 Department of Microbiology, Central Laboratory, Korle-Bu Teaching Hospital, Accra, Ghana
18 Department of Child Health, Korle-Bu Teaching Hospital, Accra, Ghana
19 Department of Public Health, Korle-Bu Teaching Hospital, Accra, Ghana
20 Department of Pharmacy, National Hospital Abuja, Abuja, Nigeria
21 Kilimanjaro Christian Medical University College, Moshi, Tanzania
22 Pharmacy Department, University Teaching Hospital, Ministry of Health, Lusaka, Zambia
23 Department of Microbiology, University Teaching Hospital, Ministry of Health, Lusaka, Zambia

Contributions

JA, NA, DE, JO, MB, TD, MMO, MF, MG, BM, CM, MC, NF, JM, KA, TAA, CHO, LB, AB, AF, BTM, NN, UC, NK, CM, and RN contributed to data collection, data checking, and project administration.
